# Language network disruption in patients with Lewy body diseases

**DOI:** 10.1007/s00702-026-03128-w

**Published:** 2026-03-07

**Authors:** Daniel Carbol, Lubomira Novakova, Patricia Klobusiakova, Irena Rektorova

**Affiliations:** 1 Applied Neuroscience Research Group, Central European Institute of Technology – CEITEC, Masaryk University, Brno, Czech Republic; 2 Faculty of Medicine, Masaryk University, Brno, Czech Republic; 3 Surgeon General Office of the Slovak Armed Forces, Ruzomberok, Slovak Republic; 4 First Department of Neurology, St. Anne’s University Hospital, Brno, Czech Republic; 5 First Department of Neurology, Faculty of Medicine, Masaryk University, Brno, Czech Republic

**Keywords:** Lewy body diseases, Mild cognitive impairment, Language network, Functional connectivity, Network efficiency

## Abstract

Lewy body diseases, including Parkinson’s disease and dementia with Lewy bodies, are marked by neuronal α‑synuclein aggregation, motor parkinsonism, cognitive impairment and diverse non‑motor symptoms including communication impairments. Compared to other symptoms, non‑motor communication impairments remain under-explored, especially outside English‑speaking cohorts. The aim of this study was to elucidate the neural underpinnings of linguistic deficits as assessed by fMRI in Czech-speaking patients diagnosed with neuronal Lewy body disease and mild cognitive impairment (LBD-MCI). Scores from the Short Neuropsychological Battery and resting‑state functional MRI data were analyzed in 26 Czech‑speaking patients with LBD‑MCI and 24 healthy controls. Analyses targeted regions of interest within the dorsal and ventral language networks. We applied graph theory metrics, within‑network connectivity and seed‑based functional connectivity analysis. Graph analysis revealed dorsal‑stream disruption in LBD‑MCI: reduced clustering coefficient, increased path length, and diminished node strength, each correlating with language functions; ventral‑stream topology remained intact. Within‑network analyses showed impaired connectivity across both pathways, but weaker coupling between the opercular inferior frontal gyrus and posterior superior temporal gyrus predicted behavioral scores in language functioning, further highlighting dorsal vulnerability. Seed‑based analysis identified reduced frontotemporal connectivity in the dorsal stream and decreased fronto‑occipital and temporo-cerebellar connections in the ventral stream. LBD‑MCI is characterized particularly by network‑specific reductions in dorsal language stream efficiency and functional connectivity, underpinning syntactic and phonological processing deficits. These findings offer novel insights in the neural basis of language impairment in LBD-MCI.

**Supplementary Information:**

The online version contains supplementary material available at https://doi.org/10.1007/s00702-026-03128-w.

## Introduction

The capacity to communicate is one of the most vital skills in humans, and its loss can profoundly impact both the individuals and their environment. In patients with Lewy body diseases (LBDs), communication impairments might not always be profoundly manifested, yet even minor deficits can significantly disrupt social interactions, ultimately diminishing the patient’s quality of life. LBDs, including Parkinson’s disease (PD) and dementia with Lewy bodies (DLB), are progressive neurodegenerative conditions marked by the accumulation of α-synuclein protein aggregates into Lewy bodies and neurites, resulting in widespread neuronal loss (Goedert et al. 2013). As the second-most prevalent cause of neurodegenerative dementia after Alzheimer’s disease, LBDs affect millions worldwide, with significant effects on motor and non-motor functions (Boström et al. 2007; Walker et al. 2015).

Non-motor deficits, most notably in the domains of language and communication, are frequently underappreciated as significant contributors to the burden experienced by patients and caregivers (Aarsland et al. 2017) and have been relatively overlooked in research. The majority of PD patients develop dementia as part of the course of the disease (Hely et al. 2008); however, the cognitive decline may already be present at the time of diagnosis, concomitant with impairments in communication (Miller 2017; Smith and Caplan 2018). In DLB, mild cognitive impairment (MCI) or dementia are present from the onset of the disease, as is their adverse impact on communication (McKeith et al. 2017). Notably, recent evidence suggests that language deficits in DLB may even precede the clinical manifestation of this broader cognitive impairment (Watanabe et al. 2023). It is clear that the communication deficits observed in both PD and DLB extend beyond motor speech challenges, such as hypokinetic dysarthria, and likely encompass difficulties in higher-order language processes. Consequently, these language difficulties can have a profound impact on patients’ social engagement and quality of life (Smith and Caplan 2018).

Language processing is a complex task requiring the integration of syntactic, semantic, and phonological information; it is heavily reliant on cognitive resources such as working memory, attention, and executive function (Caplan and Waters 2013; Yoon et al. 2015). In LBDs, these cognitive functions are frequently impaired, leading to difficulties in language processing including comprehension, production, and high-level language tasks (e.g., making inferences, comprehending ambiguous sentences and metaphors). This phenomenon may be especially true when processing syntactically complex sentences, which require substantial cognitive effort (Murray 2008).

Studies in PD have shown that patients exhibit deficits in processing complex sentences, a phenomenon tied to their reduced working memory and attention (Grossman et al. 2002, 2003; Bocanegra et al. 2015). Similarly, Hochstadt et al. (2006) and Colman et al. (2011) reported that language impairments in PD correlate with challenges in sequencing, switching, and manipulating linguistic information; these challenges are closely tied to impaired executive functioning in patients, highlighting the interplay between language and broader cognitive decline. In DLB, where cognitive impairment emerges early (McKeith et al. 2017), language deficits may be even more severe, though their nature in early-stage LBDs remains relatively underexplored.

There are two essential neural pathways supporting language processing (Saur et al. 2008; Nasios et al. 2019; Bartoň et al. 2023). The dorsal language network, which involves the posterior superior temporal gyrus, inferior parietal lobe and connections through the arcuate fasciculus and superior longitudinal fasciculus to the premotor cortex, Brodmann Area 44 and left inferior frontal cortex, is primarily responsible for phonological and syntactic processing (Friederici 2011). This network facilitates the structural analysis of linguistic stimuli, enabling the decoding of grammatical relationships (Saur et al. 2008). Conversely, the ventral language network, connecting the superior temporal gyrus and the middle temporal gyrus, inferior parietal lobe, and occipital lobe to the inferior frontal gyrus through the inferior fronto-occipital fasciculus, underpins semantic processing and comprehension (Hickok and Poeppel 2007). Both language pathways have rich, effective inter-regional connections with other brain areas and networks (Forkel and Hagoort 2024), including the visual network (particularly its occipital regions; Perrone-Bertolotti et al. 2017; Yablonski et al. 2023; Zhang et al. 2018; the frontoparietal network; Soshi 2023; and the cerebellum; Habas 2021; Jobson et al. 2024; King et al. 2022). In healthy brains, these networks and related brain regions are engaged in an orchestrated manner to support language processing. However, in LBDs, it is not entirely clear whether and to what extent neuronal Lewy body disease pathology disrupts language networks’ integrity and their inter-regional connectivity with other brain areas, potentially impairing linguistic function.

The aim of the present investigation was to examine alterations in the efficiency and connectivity of the dorsal and ventral language networks in early LBDs as compared to healthy controls (HC) and to explore their association with language functions. By examining the dorsal and ventral language networks through graph-theory-based network analysis and seed-based functional connectivity analysis, our aim was to characterize the neural underpinnings of linguistic difficulties in LBD-MCI patients. We aimed to enhance the understanding of how alterations in these networks drive language impairments in LBDs, potentially laying the groundwork for future interventions.

In accordance with the research aims, we expected that LBD-MCI patients display disrupted network topology in conjunction with altered functional connectivity within core language networks, as evidenced by graph theory metrics and seed-based analyses. Furthermore, we anticipated that resting-state fMRI reveals altered functional connectivity between language networks and other related brain areas in LBD-MCI patients. Collectively, these hypotheses suggest novel findings to facilitate a deeper understanding of the neural mechanisms that underlie language and communication impairments in LBD-MCI.

## Methodology

### Participants

Participants were recruited through the CEITEC research group database and public advertisements. Initially, 60 participants were enrolled: 27 LBD-MCI and 33 HC. Written informed consent, approved by the local ethics committee, was obtained from all participants for the conduct of the study and the subsequent publication of the results, based on the collected and anonymized data.

The inclusion criteria were as follows: right-handedness (evaluated with the Edinburgh Handedness Inventory; Oldfield 1971), Czech as the first language, age between 60 and 80 years, and a diagnosis of Parkinson’s disease and MCI (MCI-PD; as per Litvan et al. 2012) or the MCI stage of dementia with Lewy bodies (MCI-LB; as per McKeith et al. 2020). The evaluation of MCI, characterized by the presence of subjective cognitive complaints in conjunction with objective deterioration in cognitive functioning, was undertaken in accordance with Level I criteria (Goldman et al. 2015). To this end, the Montreal Cognitive Assessment was used, with a cut-off score of 26 plus a cut-off score of 1.5 standard deviations (SD) below the age-appropriate norms in any of the neuropsychological tests used. Alternatively, a patient had to score 1.5 SD below the age-appropriate norms in at least two tests. Exclusion criteria included the presence of a cardiac pacemaker or any MRI-incompatible metal implants, a history of epilepsy, diagnosed psychiatric disorders, alcohol or drug abuse, and for the HC group, the presence of LBD, other neurodegenerative disorders, or MCI/dementia.

Patients with clinically established PD were longitudinally followed by a neurologist and kept on a stable dopaminergic medication regimen for at least 4 weeks prior to baseline assessment and throughout the study. They underwent testing in the ON medication state without experiencing dyskinesias. The levodopa equivalent dose was calculated to quantify dopaminergic medication usage. The levodopa equivalent dose was determined based on established conversion factors for antiparkinsonian medications, following the methodology outlined by Tomlinson et al. (2010), with the average levodopa equivalent dose being 942.72 mg (SD = 399.65) for the whole subgroup.

Following the initial screening at Level I, which was used to categorize the HC group, the more stringent Level II criteria were also assessed as a part of the neuropsychological evaluation in the course of the study (Goldman et al. 2015). In this detailed evaluation, each cognitive domain was probed with two standardized neuropsychological tests, and impairment was defined as scoring 1.5 SD below age-adjusted norms in one or more domains. Eight individuals who were initially classified as HC by Level I criteria approached the Level II criteria (Goldman et al. 2015) for MCI and were consequently excluded from the HC group and all subsequent analyses. Additionally, 1 HC and 1 LBD-MCI participant were excluded from the study due to their inability to complete either the magnetic resonance imaging (MRI) protocol or the neuropsychological examination, resulting in missing critical data.

**Fig. 1:**
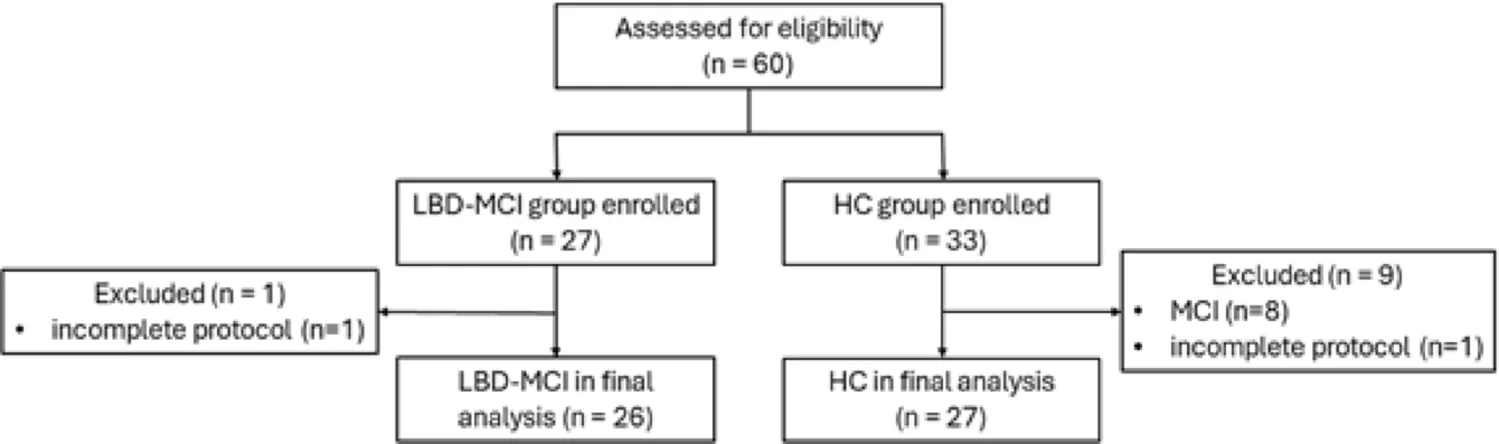
Flow diagram of the participants

The final sample comprised 26 LBD-MCI subjects (of whom 18 had been diagnosed with MCI-PD and 8 with MCI-LB) and 24 HC participants (see Fig. 1). Goldman et al. (2014) concluded that the MCI stage, being a transitional phase preceding dementia, blurs the diagnostic boundaries between Parkinson’s disease and dementia with Lewy bodies. Accordingly, MCI-PD and MCI-LB were treated as a single LBD-MCI group. Both conditions share a common alpha-synuclein–based neuropathology and exhibit highly overlapping clinical and cognitive profiles in prodromal stages (Ciafone et al. 2020).

The LBD-MCI and HC groups were statistically matched for demographic variables such as sex and education. A significant discrepancy in age was observed (see Results); consequently, the effect of age was controlled in all subsequent analyses. To ensure that the observed functional connectivity alterations were not driven by gross anatomical atrophy, we performed a control analysis of global grey matter volume (GMV; see Supplementary Text S1)

### Neuropsychological evaluation

Prior to fMRI scanning, all participants underwent a comprehensive neuropsychological examination lasting approximately 60 min, which included the Montreal Cognitive Assessment (Nasreddine et al. 2005), the Boston Naming Test (Zemanová et al. 2016), the Geriatric Depression Scale (Yesavage et al. 1982), the Functional Activities Questionnaire (Bezdicek and Lukavsky 2010), validated for the Czech population, and the Czech versions of Short Neuropsychological Battery (assessing six cognitive domains: short-term memory, visuospatial functions, language functions, attention, executive functions, and long-term memory; Straková et al. 2020).

The assessment of language functions comprised a naming task, in which patients identified objects based on verbal descriptions, and tests of verbal fluency. The latter included a semantic fluency task (naming as many animals as possible in one minute) and a phonemic fluency task (generating words beginning with the letters ‘M’ and ‘B’ within one minute). Language functions as assessed by the Short Neuropsychological Battery should rely on interactions between both language streams (Nasios et al. 2019). Specifically, in the naming task, the dorsal stream is important for transforming semantic representations into phonological forms (phonological encoding) and for motor planning stages of naming; the ventral stream supports semantic representations and the retrieval of conceptual knowledge necessary for accurate naming (McKinnon et al. 2018; Nasios et al. 2019). The category-based fluency tasks are commonly considered to have a close relationship with the ventral language pathway, as the semantic aspects of fluency performance rely heavily on accessing semantic categories and retrieving exemplar knowledge (Blecher et al. 2019). In contrast, phonemic fluency primarily recruits the dorsal stream, as it requires the strategic search and retrieval of words based on their phonological properties rather than their meaning - a process heavily dependent on the left inferior frontal gyrus and its dorsal connections (Costafreda et al. 2006; Hickok and Poeppel 2004). Additionally, the executive control required for both fluency tasks further recruits dorsal frontal circuits (Gonzalez et al. 2021). Consequently, this combination of tasks ensures that the functional integrity of both the dorsal and ventral language pathways is examined.

### MRI sequences

Data acquisition was performed using a 3 T Siemens Prisma MR scanner (Siemens Corp., Erlangen, Germany) at the Central European Institute of Technology (CEITEC), Masaryk University in Brno. The following MRI sequences were used: magnetization-prepared rapid gradient-echo high-resolution T1-weighted images (192 sagittal slices, slice thickness = 0.85 mm, TR = 2400 ms, TE = 2.27 ms, FA = 8°, FOV = 218 mm, voxel size 0.85 × 0.85 × 0.85 mm) and multiecho gradient-echo echo-planar imaging sequence (500 scans, 60 transversal slices, slice thickness = 2.5 mm, TR = 980 ms, TE = {14; 34.63; 55.26} ms, FA = 50°, FOV = 200 mm, voxel size 2.5 × 2.5 × 2.5 mm) acquired during resting-state condition (eyes closed, subjects instructed to lie still and not to fall asleep).

### Preprocessing

Resting-state fMRI data were pre-processed using MATLAB 2019a (MathWorks, Inc.) and SPM12 software. The pre-processing consisted of realignment, multi-echo data merging based on the contrast-to-noise ratio (Poser et al. 2006), normalization into standard anatomical space (Montreal Neurological Institute coordinates), and spatial smoothing. After the registration of T1 subject image to mean resliced fMRI image (coregistration), the warp (deformation) was computed for T1 subject image that best aligned the SPM MNI space template. This warp was then applied in order to register fMRI images to MNI space. Regarding distortion correction neither field mapping nor any other special method/tool was used.

We checked for motion artifacts using framewise displacement (Power et al. 2014). There were no subjects with excessive motion artifacts based on our criteria of less than 10% of scans with framewise displacement > 0.75 mm or at least one scan with framewise displacement > 3 mm. A high-pass filter with a cut‐off of 1/128 Hz was applied. The six-motion parameter time series, framewise displacement, and the signals from white matter and cerebrospinal fluid were regressed out of the data in subsequent analyses.

### Regions of interest

The analysis of the obtained fMRI data focused on specific regions of interest (ROIs) drawn from the study by Saur et al. (2008) describing dorsal and ventral brain pathways involved in language processing. For the dorsal pathway, the ROIs included the posterior superior temporal gyrus (T1p; 57, 36, 6), anterior superior temporal gyrus (T1a; 57, 3, 9), frontal operculum (FOP; 42, 27, 0), dorsal premotor cortex (PMd; 48, 0, 36), and inferior frontal gyrus (F3op; 45, 9, 24). For the ventral pathway, the ROIs comprised the posterior middle temporal gyrus (T2p; 48, 60, 18), anterior middle temporal gyrus (T2a; 51, 0, 18), fusiform gyrus (FUS; 30, 33, 18), inferior frontal gyrus pars triangularis (F3tri; 48, 27, 12), and inferior frontal gyrus pars orbitalis (F3orb; 45, 27, 12).

**Fig. 2:**
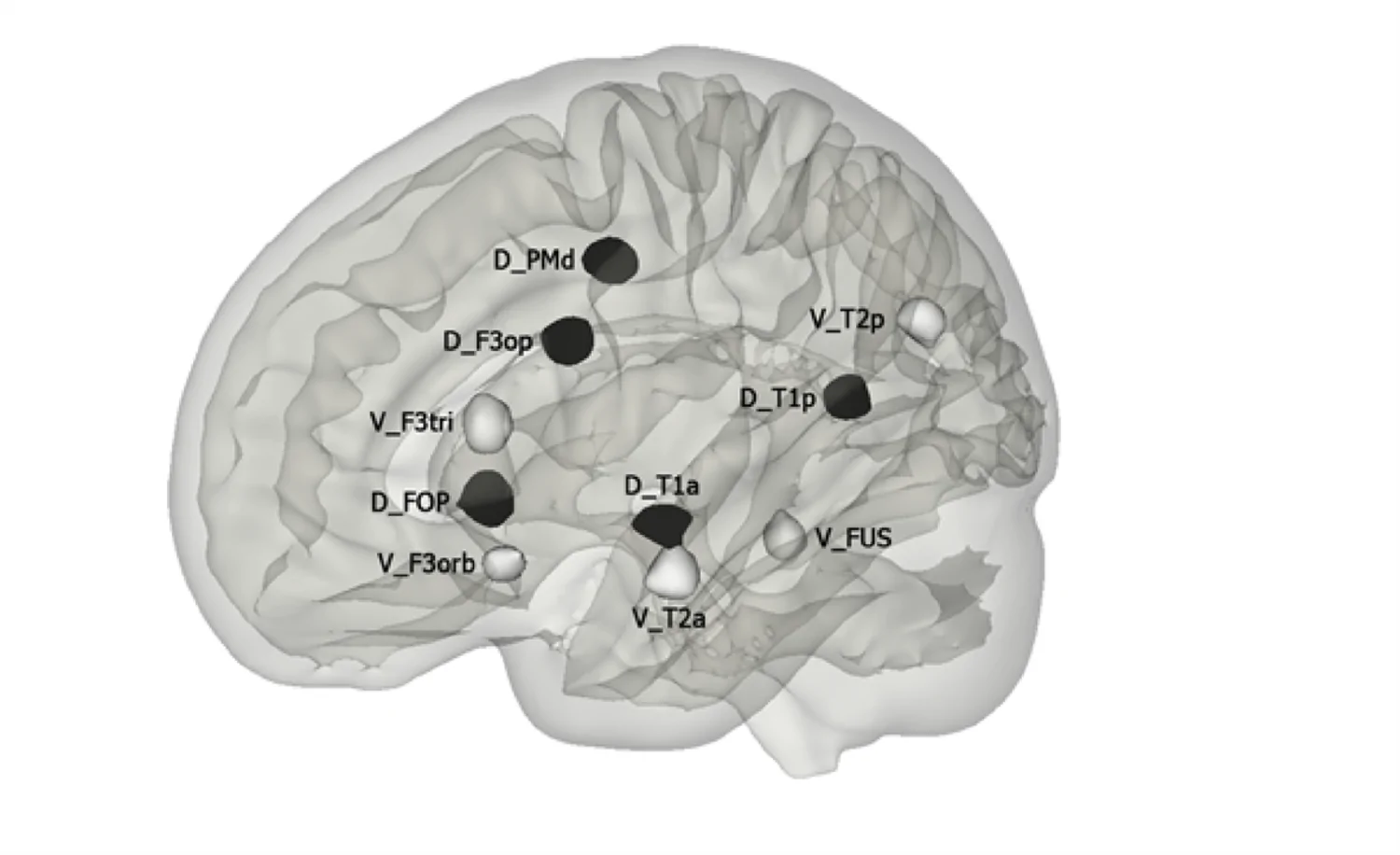
Illustration of the ROIs used in our analyses, with dorsal pathway regions indicated in light grey and ventral pathway regions indicated in dark grey

Rather than using a standard anatomical atlas, spheres with the centroid at the corresponding peak voxel (radius = 6 mm) were created and intersected with the fMRI group mask. This mask is a group mask created using mask explorer toolbox (Gajdoš et al. 2016). Firstly, individual subjects’ binary masks are calculated using the mentioned toolbox as following: a threshold of 80% of the image mean intensity is used in order to differentiate between brain tissue (value 1) and background noise (value 0). Then, a group mask is calculated as a conjunction of subjects’ masks. The final masks then served as dorsal and ventral language network ROIs and were used in the subsequent analyses. All the regions in question are depicted in Fig. 2 and listed in Table 1, which details the correspondence between these coordinate-based regions and the atlas-based parcels used for graph analysis.

### Graph metrics

Pre-processed resting-state fMRI data were further processed using MATLAB 2019a and the Brain Connectivity Toolbox (NITRC: Brain Connectivity Toolbox: Tool/Resource Info, n.d.). The whole brain was parceled into 400 regions of interest according to the 400-region Schaefer atlas (Schaefer et al. 2018). These ROIs were intersected with individual subject binary masks (described in Sect. 2.5; Gajdoš et al. 2016) to ensure high signal quality in every subject. There were 19 ROIs that had more than 50% of signal dropouts (Gajdoš et al. 2018) in more than 10% of subjects; these ROIs were excluded from subsequent analyses. The time series of each ROI was averaged and cross-correlated using Pearson’s correlation coefficient to form a correlation matrix.

**Table 1:** Selected areas within the dorsal and ventral language network ^a^

	ROI label in Schaefer atlas	MNI coordinates			Corresponding ROIs from Saur et al. (2008) ^b^	
		R	A	S		
Dorsal						
	85	−50	3	38	PMd	
	131	−45	20	27	F3op	
	186	−48	28	0	FOP	
	195	−53	6	−11	T1a	
	198	−52	−43	5	T1p	
Ventral						
	128	−48	35	10	F3tri	
	170	−57	−9	−14	T2a	
	173	−45	−58	21	T2p	
	185	−46	32	−10	F3orb	
	193	−30	−33	−18	FUS	

^a^ The table presents the ROIs from the Schaefer atlas (2018) that were used in our analyses with their Montreal Neurological Institute coordinates (centroids) based on the final masks and corresponding area labels used by Saur et al. (2008).

^b^ PMd - dorsal premotor cortex, F3op - opercular part of the inferior frontal gyrus, FOP - frontal operculum, T1a - anterior superior temporal gyrus, T1p - posterior superior temporal gyrus, F3tri - triangular part of the inferior frontal gyrus, T2a - anterior middle temporal gyrus, T2p - posterior middle temporal gyrus, F3orb - orbital part of the inferior frontal gyrus, FUS - fusiform gyrus.

(381 × 381) for each subject. For further analyses, Fisher’s z-transformed Pearson’s correlation coefficients were used. Weighted networks were analyzed so that useful information about the strength of connections was preserved. Negative correlations were replaced with zeros.

We computed the following global metrics: average clustering coefficient, characteristic path length, average node strength, modularity, and global efficiency. The local clustering coefficient, nodal path length, and node strength were calculated for ROIs of the whole brain. Next, the average value of each measure was computed specifically for the dorsal and ventral language network as a representative local measure. The selection of specific parcels from the Schaefer atlas that correspond to the dorsal and ventral language networks defined by Saur et al. (2008) is detailed in Table 1.

### Seed-based analysis

Seed-based functional connectivity analysis was performed on pre-processed resting-state data. Specifically, the abovementioned final masks serving as dorsal and ventral language network ROIs (see Saur et al. 2008) were generated and their corresponding mean time series were then extracted and entered in the SPM first-level model as a regressor. The resulting contrasts were then entered into a two-sample t-test to identify brain areas with connectivity differences between LBD-MCI and HC groups, with age used as covariate of no interest. Clusters that survived an initial voxel-wise threshold of *p* <.001 (uncorrected) and a cluster-level FWE correction at *p* <.05 were defined as significant.

For post-hoc characterization of these effects, mean beta values from their first-level seed-to-voxel maps were extracted for each participant and averaged within these significant clusters, resulting in one average connectivity value for each seed-cluster pair. These per-subject average connectivity values were compared between the LBD-MCI and HC groups using Analysis of Covariance (ANCOVA) with age as a covariate. To ensure robustness against non-normal distributions, parameter estimates were calculated using bias-corrected and accelerated (BCa) bootstrapping with 1000 resamples. The overall statistical threshold was set at *p* <.05 FWE-corrected at the cluster level, with an initial voxel-wise cut of *p* <.001 uncorrected.

### ROI analysis (dorsal/ventral LN)

In order to analyze resting-state functional connectivity between individual ROIs in dorsal and ventral language network separately, the time series of each ROI (abovementioned final sphere masks; Saur et al. 2008) were averaged and cross-correlated using Pearson’s correlation coefficient. Thus, a correlation matrix for each subject for dorsal and ventral language network separately was created and Fisher’s z-transformed. Z values then served as representative resting-state connectivity measures.

### Statistical analysis

All statistical analyses were performed using software from MATLAB and SPSS. Group comparisons between LBD-MCI and HC in demographic variables not accounting for age were performed using the Mann-Whitney U test. The χ^2^ test was used to compare categorical variables such as sex. For neuropsychological domains, graph metrics and connectivity metrics, group differences between LBD-MCI and HC were assessed using Analysis of Covariance (ANCOVA) with age included as a covariate. To account for the non-normal distribution of the data, significance was determined using bias-corrected and accelerated (BCa) bootstrapping with 1000 resamples; we also tested group × age interactions to verify the homogeneity of regression slopes. To ensure that the observed significant between-group differences were not influenced by demographic variables or depressive symptoms, we performed a sensitivity analysis using ANCOVA models that included Sex, Education, and GDS as additional covariates.

In order to further investigate the roles of specific language networks, an additional correlation analysis was performed, focusing on network-specific graph theory metrics and resting-state connectivity values for both the dorsal and ventral language networks and their associations with neuropsychological domains. In addressing the association between cognitive performance, language functions, graph metrics and seed-to-seed connectivity, partial correlations were employed, with the effect of age being controlled for. The subsequent objective was to determine the predictive capacity of both the language network efficiency disruption and the connectivity disruption on behavioral scores of language function. To this end, two hierarchical linear regression analyses were performed to determine the predictive value of network alterations while controlling for the effect of age. In the first analysis, language functions were designated as the dependent variable; age was entered in the first block, while dorsal and ventral network graph metrics were entered stepwise in the second block. In the second analysis, the same hierarchical procedure was applied, with seed-to-seed connectivity values defined as the independent variables in the second step.

## Results

### Sample characteristics

The LBD-MCI group was, on average, 6.2 years older than the HC group (*p* <.05). The LBD-MCI group achieved significantly lower scores than the HC group in language functions and in all tested domains except for attention.

**Table 2:** Characteristics of the sample

Factor	LBD-MCI ^a^	HC	Test statistics of the between-group difference ^a^		
N	26	24			
Participant characteristics					
Sex			*X*^2^ (1, *N* = 50) = 0.28		
Female (n)	10	12			
Male (n)	15	13			
Age (M ± SD ^b^)	72.31 (± 5.65)	66.08 (± 3.98)	U = 109, Z = −3.95*		
Education length ^c^ (M ± SD)	15.04 (± 3.87)	16.33 (± 2.76)	U = 246.5, Z = −1.29		
Grey matter volume proportion (M ± SD	0.37 (± 0.03)	0.4 (± 0.03)	F (1,47) = 1.2, η^2^_p_ = 0.03		
Neuropsychological examination (M ± SD)					
Montreal Cognitive Assessment score	23.38 (± 2.56)	27.25 (± 1.36)	F (1,47) = 25.2, η^2^_p_ = 0.35*		
Short-term memory z-score	−1.12 (± 0.81)	− 0.07 (± 0.76)	F (1,47) = 13.34, η^2^_p_ = 0.22*		
Visuospatial functions z-score	−0.03 (± 0.5)	0.65 (± 0.54)	F (1,47) = 14.92, η^2^_p_ = 0.24*		
Attention z-score	−0.76 (± 0.67)	−0.3 (± 0.77)	F (1,47) = 1.16, η^2^_p_ = 0.02		
Executive functions z-score	−1.28 (± 0.7)	−0.13 (± 0.64)	F (1,47) = 19.35, η^2^_p_ = 0.29*		
Long-term memory z-score	−1.42 (± 0.64)	−0.51 (± 0.6)	F (1,47) = 14.89, η^2^_p_ = 0.24*		
Language functions z-score	−0.73 (± 0.68)	0.14 (± 0.4)	F (1,47) = 12.12, η^2^_p_ = 0.21*		
Geriatric Depression Scale score	3.08 (± 3.07)	0.87 (± 1.15)	F (1,47) = 5, η^2^_p_ = 0.1*		
Functional Activities Questionnaire score	2.54 (± 4.29)	0 (± 0)	F (1,47) = 4.2, η^2^_p_ = 0.08*		

^a^ Due to the non-normal distribution of most variables, group comparisons were performed using either non-parametric tests (Mann-Whitney U) or ANCOVA with age included as a covariate. For ANCOVA, parameter estimates were calculated using bias-corrected and accelerated (BCa) bootstrapping with 1000 resamples.

^b^ Values in the table represent unadjusted means ± standard deviation.

^c^ Education length is quantified as the total number of years of formal education completed.

* The difference is significant at the *p* <.05 level after applying the Holm-Bonferroni FWE correction

We assessed global grey matter volume to control for potential atrophy-related confounds. After controlling for age, no significant group differences in GMV were observed and structural volume did not correlate with functional connectivity metrics (for full details and statistics, see Supplementary Text S1). Consequently, age was included as the sole nuisance covariate in all statistical models. For a detailed comparison of the groups, please refer to Table 2.

To validate the combination of MCI-PD and MCI-LB patients into a single LBD-MCI cohort, we performed a comprehensive comparison of these subgroups. the only significant between-subgroup difference was observed on the Functional Activities Questionnaire (U = 29,5, Z = −2.363, *p* =.032). On average, MCI-PD patients scored 3.125 points lower on the 30-point scale, indicating slightly reduced independence in instrumental activities of daily living compared with MCI-LB patients. No other significant differences were found in demographics, neuropsychological performance (see Supplementary Table S2 for a detailed comparison of the MCI-PD and MCI-LB subgroups), graph metrics or connectivity measures. Furthermore, a sensitivity analysis excluding the MCI-LB subgroup yielded results consistent with the primary analyses. Consequently, the LBD-MCI sample was treated as a single group for the purposes of the present analyses.

### Graph analysis

Graph theory analysis demonstrated significant disruptions in global network efficiency among LBD-MCI relative to HC. ANCOVA tests controlling for age disclosed that LBD-MCI patients exhibited a significantly lower average global clustering coefficient [F(1,47) = 6.23, η^2^_p_ = 0.12, *p* =.016] and global node strength [F(1,47) = 6.81, η^2^_p_ = 0.13, *p* =.012], alongside a greater average global path length [F(1,47) = 7.87, η^2^_p_ = 0.14, *p* =.007], compared to HC.

To delineate network-specific effects, graph analyses were conducted separately for the dorsal and ventral language networks. Within the dorsal language network, significant alterations were observed in LBD-MCI patients, with a decreased clustering coefficient [F(1,47) = 6.16, η^2^_p_ = 0.12, *p* =.017], an elevated average path length [F(1,47) = 8.58, η^2^_p_ = 0.15, *p* =.005] and a reduced node strength [F(1,47) = 6.86, η^2^_p_ = 0.13, *p* =.012]. The between-group differences persisted after covarying for Sex, Education, and GDS scores. In contrast, parallel analysis of the ventral language network revealed no significant differences in these graph metrics between LBD-MCI and HC.

With regard to the associations observed with neuropsychological domains, the correlational analysis indicated a significant correlation between the metrics and language function, especially within dorsal network. Nevertheless, a distinction was identified between the two networks: metrics from the dorsal language network were also correlated with executive function and long-term memory; those from the ventral language network demonstrated correlations with attention. The detailed results of this analysis are presented in Table 3.

In terms of the regression analysis investigating the graph metrics disruption as a predictor of language functions decline, when controlled for age, the model proved to be significant, F(1,48) = 5.735, *p* =.021, explaining 10.7% of the variance beyond what age explained (R² = 0.107). The metric of dorsal path length was a sole significant negative predictor of language function z-score (B = − 0.370, SE = 0.154, β = − 0.327, t = − 2.4, *p* =.021).

**Table 3:** Correlations between network-specific graph metrics and examined neuropsychological domains

Variable	Dorsal clustering coefficient	Dorsal path length	Dorsal node strength	Ventral clustering coefficient	Ventral path length	Ventral node strength	
Language functions	0.316*	− 0.367*	0.319*	0.267	− 0.315*	0.247	
Short-term memory	0.181	− 0.227	0.165	0.170	− 0.232	0.151	
Visuospatial functions	0.142	− 0.157	0.144	0.086	− 0.131	0.073	
Attention	0.261	− 0.266	0.276	0.285*	− 0.286*	0.319*	
Executive functions	0.301*	− 0.320*	0.301*	0.261	− 0.296*	0.231	
Long-term memory	0.325*	− 0.332*	0.347*	0.260	− 0.265	0.251	

* *p* <.05

### Within-network seed-based analysis

From a total of 20 pre-specified pairwise combinations examined − 10 within the dorsal language network (comprising PMd, F3op, FOP, T1a, and T1p) and 10 within the ventral language network (comprising F3tri, T2a, T2p, F3orb, and FUS) - four connections exhibited statistically significant reductions in LBD-MCI as compared to HC. Specifically, within the dorsal language network, connectivity between the opercular part of the inferior frontal gyrus (F3op) and the posterior superior temporal gyrus (T1p) was markedly diminished in LBD-MCI [F(1,47) = 9.41, η^2^_p_ = 0.17, *p* =.004]. This link within the dorsal network was significantly correlated with language functions (*r* =.283; *p* =.04). Including Sex, Education, and GDS scores did not alter the overall pattern of findings.

Within the ventral language network, significant reductions were observed in connectivity between the triangular part of the inferior frontal gyrus (F3tri) and the anterior middle temporal gyrus [T2a; F(1,47) = 6.36, η^2^_p_ = 0.12, *p* =.015]. However, this link was not correlated with language functions. There was also a significant reduction in connectivity between F3tri and the posterior middle temporal gyrus (T2p; F(1,47) = 5.45, η^2^_p_ = 0.1, *p* =.024], yet again without correlation with the language function. Lastly, the connection was reduced between T2a and T2p [F(1,47) = 5.44, η^2^_p_ = 0.1, *p* =.024], also with no significant correlation with language function. Sensitivity analysis adjusting for sex, education length, and GDS scores failed to confirm the robustness of group difference for the T2a–T2p functional connection (*p* =.051). In fully adjusted model, neither the group effect nor any of the additional covariates reached statistical significance, suggesting that the originally observed difference was likely driven by shared variance among demographic and clinical factors rather than a specific disease effect. It is also important to note that none of the within-network connectivity differences retain their significance after applying a multiple-comparisons correction. For the complete list of all pairwise comparisons, see Supplementary Table S3.

With regard to the regression analysis, the investigation into the potential predictive capacities of resting-state connectivity impairments within language networks on behavioral language functioning, when controlled for age, yielded a significant model (F(1,47) = 4.1, *p* <.04). The model accounted for 6.4% of the variance above that explained by age (R² = 0.064). The value of the resting-state connectivity between the opercular part of the inferior frontal gyrus (F3op) and the posterior superior temporal gyrus (T1p) emerged as a significant positive predictor of language function z-score (B = 1.269, SE = 0.627, β = 0.253, t(47) = 2.025, *p* =.04).

### Whole-brain seed-based analysis

In LBD-MCI patients as compared to HC, the seed-based analysis of resting-state functional connectivity revealed significant reductions in the connectivity of the language network ROIs with other areas in the rest of the brain. As illustrated in Table 4, significant differences were identified between the LBD-MCI group and the HC group for multiple seed-cluster pairs. The results remained stable after adjusting for Sex, Education, and GDS scores. Of the connections showing significant group differences, three involving dorsal network nodes and two involving ventral network nodes exhibited significant correlations with language function.

**Table 4:** Reduced functional seed-to-seed connectivity in LBD-MCI patients compared to healthy controls

Seed region ^a^		Target region^b^	U	Z	p	r^c^
Dorsal Language Network						
F3op		left superior temporal gyrus	163	−2.893	0.004	0.347*
F3op		bilateral cuneus	99	−4.136	< 0.001	0.288*
T1p		left superior and middle frontal gyrus	130	−3.534	< 0.001	0.437*
T1p		left cerebellum (declive)	139	−3.359	< 0.001	0.241
Ventral Language Network						
F3tri		left middle occipital gyrus	166	−2.835	0.005	0.379*
T2a		bilateral middle occipital gyrus, cuneus and precuneus	54	−5.01	< 0.001	0.268
T2p		left cerebellum (declive, culmen)	129	−3.554	< 0.001	0.258
T2p		right cerebellum (declive, cerebellar tonsil)	117	−3.787	< 0.001	0.228
T2p		right caudate and putamen	108	−3.961	< 0.001	0.237
T2p		left superior temporal gyrus and putamen	167	−2.816	0.005	0.488**

^a^ The seed region shows the starting point of the connectivity analysis.

^b^ The target region denotes the brain region with which the seed region shows reduced connectivity in LBD-MCI patients.

^c^ The *r* column shows partial correlations of connections with language functions, age controlled.

* *p* <.05 and ** *p* <.001.

In summary, the ROIs of the dorsal language network demonstrated significant decreases in left-sided frontotemporal connectivity, connectivity with the left posterior cerebellum (declive) and the bilateral cuneus. In the ventral network ROIs, a reduction in connectivity was observed, particularly with occipital regions, the posterior and anterior parts of the cerebellum (declive with culmen in the left hemisphere and declive with cerebellar tonsil in the right hemisphere), the right caudate nucleus, and the left superior temporal gyrus.

## Discussion

The present study examined language processing deficits in Czech-speaking patients with LBD-MCI, highlighting the interplay between language functioning, network efficiency, and functional connectivity alterations in the dorsal and ventral language networks. Graph theory analysis indicated particularly impaired efficiency and integration in the dorsal language network. The results of the functional connectivity analyses revealed disruptions across both networks, while only a single dorsal connectivity metric emerged as a predictor of language functioning. The findings of this study indicate that the observed variations in language functions among the study groups can be partially attributable to these dorsal network differences. The findings focus specifically on the neural properties of language networks and serve to supplement and expand upon the results of earlier studies examining language processing and production in patients with PD and LBD. These earlier studies demonstrated that patients with LBDs experience progressive deterioration in brain connectivity, with the result that both their motor and cognitive functions are impacted in the context of language processing (Cotelli et al. 2007; Péran et al. 2009). The pattern of broad disruption in language networks and its association with various cognitive domains uncovered in this study is also consistent with earlier resting-state fMRI studies in LBDs, which have reported connectivity changes associated with cognitive decline (Baggio et al. 2014; Peraza et al. 2014).

Graph theory analysis revealed significant reductions in network efficiency in LBD-MCI patients, particularly within the dorsal language network, which is critical for phonological and syntactic processing. The path length showed the strongest association with behavioral measures of language functioning and emerged as a significant negative predictor. In contrast, the ventral language network, responsible for semantic processing, showed no significant disruptions in any of the network efficiency properties. These findings highlight a selective disruption of language network properties in LBD-MCI. This could potentially reflect disease-specific impairments in the neural architecture supporting phonological and syntactic processing (Friederici 2011), suggesting its vulnerability, while the ventral network’s global organization appears relatively preserved.

The correlational analysis further showed that apart from association with language functions, the dorsal network’s metrics also correlated with executive functions and long-term memory. The dorsal network efficiency and executive functioning were both diminished in LBD-MCI; the correlation between these might suggest that syntactic processing encompasses higher-order executive processes. Therefore, it could be hypothesized that the decline in executive functioning gives rise mainly to the syntactic aspect of linguistic difficulties. Similarly, the association with long-term memory implies that the dorsal network supports the retrieval of associations, patterns or schemas, further contributing to linguistic abilities (Caplan and Waters 2013). Overall, these findings may explain why LBD-MCI patients exhibit particular linguistic difficulties such as comprehension and naming deficits (Murray 2008), as these tasks demand both executive resources and memory retrieval – areas often impaired early in LBDs (Aarsland et al. 2017). Problematic syntactic processing is also supported by studies in PD or MCI-LB showing difficulties with complex sentence structures (Hochstadt et al. 2006; Novakova et al. 2023, 2025). Furthermore, this pattern of dorsal stream vulnerability mirrors the network degeneration observed in non-fluent/agrammatic primary progressive aphasia (Henry et al. 2016). While LBD-MCI is clinically distinct from PPA, this shared anatomical disruption likely underpins the syntactic difficulties observed in both conditions, implying a link between dorsal tract integrity and grammatical processing (Mandelli et al. 2016).

The identification of within-network frontotemporal connectivity disturbances provides insight as to which segments of the language networks are most affected. The connectivity between the inferior frontal gyrus and the posterior superior temporal gyrus appears to be the most negatively impacted within the dorsal network; in the ventral network, the connections between the triangular part of the inferior frontal gyrus, the anterior middle temporal gyrus, and the posterior middle temporal gyrus appear to be weakened. However, the regression analysis demonstrated that only the connectivity within the dorsal network significantly predicts scoring in behavioral measures of language functioning. Consequently, the seed-based connectivity analysis yielded further evidence of widespread network alterations in LBD-MCI, unveiling diminished functional connectivity and its potential influence on language deficits – particularly with regard to the effect of frontotemporal connectivity within the dorsal language network. This finding further corroborates the aforementioned results, indicating disrupted neural communication within the dorsal network.

The observed reductions in dorsal network whole-brain resting-state connectivity with other key language-related regions are consistent with previous studies highlighting the critical role of these regions in syntactic processing and phonological working memory (Friederici 2011). In particular, the frontotemporal connectivity in the dorsal language pathway, such as the opercular part of the inferior frontal gyrus (F3op; a part of Broca’s area) and the left superior temporal gyrus, is hypothesized to facilitate top-down processes that are instrumental in the determination of grammatical relations (Friederici 2011). The diminished connectivity between the posterior superior temporal gyrus (T1p) and regions including the left superior frontal gyrus and the cerebellum might suggest a broader disruption in the integration of linguistic and executive mechanisms, as the T1p is traditionally considered a neurological basis for language comprehension (Buchsbaum et al. 2001; Turken and Dronkers 2011), while the left superior frontal gyrus is linked to higher cognitive and executive functions (du Boisgueheneuc et al. 2006). The cerebellum is traditionally linked to motor aspects of language deficits (De Smet et al. 2007); however, it has been increasingly recognized for its role in language processing, particularly in coordinating lexical retrieval and syntactic structuring (Murdoch 2010; Highnam and Bleile 2011; Mariën et al. 2014). van Dun et al. (2016) argued that the cerebellum is involved in language in a similar manner as it is involved in motor functions – through monitoring/coordinating cortical functions. Nasios et al. (2019) stated that the cerebellum ought to be included in an updated “multiple stream model” of language processing, as its targeting with transcranial direct current stimulation proved to have enhancing effects on verbal fluency (Turkeltaub et al. 2016). Thus, reduced connectivity with the cerebellar subparts – such as the declive, culmen, and cerebellar tonsils – may at least partially underlie deficits in language processing in LBD-MCI.

With regard to the ventral network, which is principally responsible for semantic processing and lexical access (Hickok and Poeppel 2004, 2007), between-network connectivity was also disrupted. This was particularly evident in the anterior part of the middle temporal gyrus (T2a), which exhibited significantly reduced connectivity with the middle occipital gyrus and precuneus. The middle temporal gyrus has been identified as a crucial hub for semantic memory and control with a role in accessing and integrating word meanings (Binder and Desai 2011; Ralph et al. 2017); the precuneus activation has also been observed in processing sentence semantics (Price 2010), particularly those involving spatial linguistic information and relations (Wallentin et al. 2008). However, it is important to note that unlike the dorsal network findings, these specific ventral connectivity reductions did not correlate with language function scores in our sample. While the reduced connectivity implies a compromise in the neural architecture supporting conceptual retrieval and spatial integration (Grossman et al. 2002), the lack of behavioral association suggests that these deficits may not yet be the critical limiting factor for language performance at this disease stage. The diminished connection between the posterior part of the middle temporal gyrus (T2p) and the cerebellum then points to a similar pattern of disrupted connectivity as the one observed in the dorsal network, indicating that specific cerebellar subparts might play a coordinating role (van Dun et al. 2016) in the semantic aspect of language processing as well as in the syntactic one. Yet the absence of a correlation with language scores further indicates that the observed communication deficits are predominantly driven by dorsal stream dysfunction.

Several limitations should be noted when interpreting the findings of this study. The relatively small sample size may constrain statistical power and limit the ability to generalize the results to a broader population. We addressed this by employing rigorous diagnostic criteria to ensure sample homogeneity and by using bootstrapping to ensure statistical robustness. Second, while we utilized standardized tests to assess language function, these tasks inevitably recruit domain-general cognitive processes such as attention and executive control. The correlations observed between dorsal network metrics and executive function scores likely reflect the intrinsic functional overlap of these domains – specifically, the dorsal stream’s role as an interface between syntactic processing and executive resources – rather than a lack of test specificity. Future studies with larger cohorts and more granular linguistic tasks are needed to further disentangle these components. Furthermore, the potential influence of medications used by participants presents another challenge; while patients were on stable regimens, we cannot fully rule out the possibility that these medications affected cognitive or language performance, potentially confounding the results. Additionally, a limitation of this study is the pooling of MCI-PD and MCI-LB patients into a single LBD-MCI cohort. The clinical distinction between these diagnoses is largely operational and does not necessarily reflect a clear biological separation, resulting in highly overlapping features at the MCI stage. Accordingly, our supplementary analyses confirmed the subgroups were comparable across all demographic, cognitive, and network measures, differing only in Functional Activities Questionare. Nevertheless, we acknowledge that subtle clinical differences, such as motor symptom burden, could influence resting-state dynamics or task execution in ways that might be obscured when the diagnostic subgroups are combined. And finally, the patient group was older than the HC group, which could influence the outcomes. We tried to limit this potential influence by adjusting for the effect of age in the analyses. These limitations highlight the need for careful consideration of the results and suggest directions for future studies to address these gaps.

In essence, these findings contribute to the understanding of language impairments in LBDs, extending beyond motor speech deficits such as hypokinetic dysarthria (Smith and Caplan 2018). The graph theory and seed-based analyses of resting-state fMRI data revealed significant disturbances in both the efficiency and connectivity of the dorsal language network, significantly correlating with language functioning. Notably, the path length metric and within-network connectivity between F3op and T1p areas predicted the scores in language processing tasks. Thus, the selectively impaired dorsal network properties suggest that syntactic processing may be particularly vulnerable in early-stage LBDs. The additional involvement of the ventral network connectivity indicates that semantic processing is also altered (Goedert et al. 2013), although its impact on certain language processing tasks might not be behaviorally manifested. The whole-brain connectivity analyses further confirm the findings of language network deficiency, confirming the inter-regional connectivity disturbances with other language-related brain areas. These results have the potential to enhance the current understanding of the underlying mechanisms that lead to language impairments in LBDs, thereby paving the way for the development of future intervention strategies.

## Supplementary Information

Below is the link to the electronic supplementary material.

Supplementary Material 1

## Data Availability

The data that support the findings of this study are available on request from the corresponding author. The data are not publicly available due to privacy or ethical restrictions.
