# Supplementary material for: Language network disruption in patients with Lewy body diseases
Source: J Neural Transm (Vienna). 2026 Mar 7;133(8):1903–16. doi: 10.1007/s00702-026-03128-w (PMC13545036; doi:10.1007/s00702-026-03128-w)
Supplement: Supplementary file 1 [file 702_2026_3128_MOESM1_ESM.docx]

**Supplementary Materials**

**Supplementary Text S1**

To assess potential confounding effects of global grey-matter volume on our functional and behavioral outcomes, we derived a GMV/TIV ratio from the native-space VBM tissue segmentations. For each subject, GM values from the unregistered and unsmoothed GM segmentation were integrated over all voxels and divided by total intracranial volume (TIV; sum of GM, WM, and CSF values). The resulting unitless GMV/TIV proportion was used in subsequent analyses to evaluate whether global atrophy influenced the reported findings. SPM12 software was used to segment anatomical T1-weighted images.

In regards of the GMV/TIV in the LBD-MCI group and the HC, there was a significant difference between the groups. Patients showed lower GMV/TIV than controls, but GMV/TIV was strongly associated with age (r = .65), consistent with normative age-related grey-matter decline (e.g., Fjell & Walhovd, 2010). An ANCOVA controlling for age rendered the group difference non-significant and partial correlations controlling for age showed no associations between GMV/TIV and functional connectivity, language performance, or graph metrics. Because GMV/TIV did not explain variance beyond age, it was not included as an additional covariate in subsequent analyses. Therefore, age was included as the sole nuisance covariate in all statistical models. For a detailed comparison of the groups, please refer to Table 2.

**Supplementary Table S2**

| **Table S2** *Demographic and Clinical Comparison between MCI-PD and MCI-LB subgroups* | | | | |  |
| --- | --- | --- | --- | --- | --- |
| Factor | | | MCI-PD | MCI-LB | Test statistics of the between-group difference ^b^ |
| N | | | 18 | 8 |  |
| Participant characteristics | | |  |  |  |
|  | Sex | |  |  | *X*^2^ (1, N = 26) = 2.82 |
|  |  | Female (n) | 5 | 3 |  |
|  |  | Male (n) | 13 | 5 |  |
|  | Age (M ± SD) | | 71.72 (± 6.02) | 73.63 (± 4.81) | U = 62, Z = -0.56 |
|  | Education length (M ± SD) ^a^ | | 15.44 (± 3.85) | 14.13 (± 4.02) | U = 61.5, Z = -0.59 |
|  | Grey matter volume proportion | | 0.36 (± 0.04) | 0.37 (± 0.03) | U = 51, Z = -1.16 |
| Neuropsychological examination (M ± SD) | | |  |  |  |
|  | Montreal Cognitive Assessment score | | 23.44 (± 2.75) | 23.25 (± 2.25) | U = 68, Z = -0.22 |
|  | Short-term memory z-score | | -1.2 (± 0.79) | -0.94 (± 0.88) | U = 51, Z = -1.17 |
|  | Visuospatial functions z-score | | -0.05 (± 0.5) | 0.02 (± 0.53) | U = 55, Z = -0.94 |
|  | Attention z-score | | -0.74 (± 0.72) | -0.8 (± 0.58) | U = 68.5, Z = -0.19 |
|  | Executive functions z-score | | -1.34 (± 0.73) | -1.14 (± 0.63) | U = 48, Z = -1.33 |
|  | Long-term memory z-score | | -1.43 (± 0.72) | -1.4 (± 0.47) | U = 58, Z = -0.78 |
|  | Language functions z-score | | -0.89 (± 0.84) | -0.56 (± 0.48) | U = 50, Z = -1.22 |
|  | Geriatric Depression Scale score | | 3.13 (± 2.7) | 2.5 (± 3.93) | U = 38, Z = -1.94 |
|  | Functional Activities Questionnaire score | | 3.5 (± 4.88) | 0.38 (± 0.52) | U = 29.5, Z = -2.36* |
| ^a^ Education length is quantified as the total number of years of formal education completed.  ^b^ Due to the small sample sizes and distributions of the variables that do not meet the assumption of normality, non-parametric tests were performed; residualization procedure was applied to account for potential age-related confounding (the scores were regressed on age using a linear model, and the unstandardized residuals, reflecting age-independent variance, were extracted). | | | | | |
| * The difference is significant at the p < .05 level after adjusting for the effect of age and applying the Holm-Bonferroni FWE correction. | | | | | |

**Supplementary Table S3**

| **Table S3** | | | | | |  |
| --- | --- | --- | --- | --- | --- | --- |
| *Within-network connectivity differences between LBD-MCI patients and healthy controls* | | | | | | |
| **Seed Pair** | |  | F (1,47) | η^2^_p_ | p | r^a^ |
| Dorsal Language Network | | | | | |  |
|  | F3op – FOP |  | 1.49 | 0.03 | .229 | 0.02 |
|  | F3op – PMd |  | 0.60 | 0.01 | .441 | 0.27 |
|  | F3op – T1a |  | 3.19 | 0.06 | .081 | -0.14 |
|  | F3op – T1p |  | 9.41 | 0.17 | .004 | 0.28* |
|  | FOP – PMd |  | 0.64 | 0.01 | .428 | -0.12 |
|  | FOP – T1a |  | 0.29 | 0.01 | .596 | -0.12 |
|  | FOP – T1p |  | 2.03 | 0.04 | .161 | 0.37* |
|  | PMd – T1a |  | 2.70 | 0.05 | .107 | 0.05 |
|  | PMd – T1p |  | 1.58 | 0.03 | .214 | 0.2 |
|  | T1a – T1p |  | 0.00 | 0.00 | .953 | 0.31* |
| Ventral Language Network | | | | | |  |
|  | F3orb – F3tri |  | 0.41 | 0.01 | .524 | 0.16 |
|  | F3orb – FUS |  | 0.41 | 0.01 | .527 | 0.25 |
|  | F3orb – T2a |  | 0.29 | 0.01 | .594 | 0.22 |
|  | F3orb – T2p |  | 0.26 | 0.01 | .612 | 0.19 |
|  | F3tri – FUS |  | 0.58 | 0.01 | .451 | -0.04 |
|  | F3tri – T2a |  | 6.36 | 0.12 | .015 | 0.24 |
|  | F3tri – T2p |  | 5.45 | 0.10 | .024 | 0.16 |
|  | FUS – T2a |  | 1.88 | 0.04 | .177 | 0.08 |
|  | FUS – T2p |  | 2 | 0.04 | .164 | -0.01 |
|  | T2a – T2p |  | 5.46 | 0.11 | .024 | 0.13 |
|  | ^a^ The *r* column shows partial correlations of connections with language functions, age controlled. * p < 0.05 | | | | |  |
